# Evaluation der COVID-19-Impfung nach breiter Anwendung – ein Zwischenfazit für Deutschland im Juli 2022

**DOI:** 10.1007/s00103-022-03618-8

**Published:** 2022-11-10

**Authors:** Anette Siedler, Viktoria Schönfeld, Caroline Peine, Nita Perumal, Amelie Friedsam, Anna Stoliaroff-Pépin, Thomas Harder

**Affiliations:** Fachgebiet Impfprävention, Robert Koch-Institut, Seestr. 10, 13353 Berlin, Deutschland

**Keywords:** COVID-19, SARS-CoV‑2, Impfquoten, Wirksamkeit, COVID-19, SARS-CoV‑2, Vaccine coverage, Effectiveness

## Abstract

Seit Dezember 2020 stehen in Deutschland Impfstoffe gegen COVID-19 zur Verfügung. Zu den Hauptaufgaben des Fachgebiets Impfprävention des Robert Koch-Instituts (RKI) in der COVID-19-Pandemie gehören die Erhebung von Impfquoten und das Monitoring der Wirksamkeit der Impfung. Der Artikel gibt einen Überblick über die hierfür während der Pandemie etablierten Strukturen, Datengrundlagen und Studien. Ausgehend vom Digitalen Impfquotenmonitoring (DIM), welches für die tagesaktuelle Berechnung der Impfquote in mehreren Altersgruppen verwendet wird, werden die Berechnung der Inzidenzen nach Impfstatus und die Methodik der Impfeffektivitätsschätzung gegen verschiedene Endpunkte (Hospitalisierung, intensivstationäre Betreuung, Tod) beschrieben. Während diese Methode lediglich eine erste Abschätzung der Impfeffektivität erlaubt, kann in bevölkerungsbezogenen nichtrandomisierten Studien eine detailliertere und genauere Untersuchung der Wirksamkeit der COVID-19-Impfstoffe unter Realbedingungen erfolgen. Hierzu wird die gemeinsam mit dem Paul-Ehrlich-Institut (PEI) durchgeführte krankenhausbasierte Fall-Kontrollstudie COViK vorgestellt. Die Vorteile und Limitationen der genannten Strukturen und Instrumente werden diskutiert. Abschließend wird ein Ausblick auf hieraus resultierende künftige Herausforderungen in der Pandemie und beim Übergang in die endemische Lage gegeben.

## Abstract (en)

Vaccines against COVID-19 have been available in Germany since December 2020. At the Robert Koch Institute (RKI), the Immunization Unit is responsible for monitoring vaccination coverage and assessment of vaccine effectiveness. This article provides an overview of the respective reporting structures, vaccination databases, and epidemiological studies established by the Immunization Unit during the COVID-19 pandemic. We describe the COVID-19 Digital Vaccination Coverage Monitoring (DIM), which provides daily updates on vaccination coverage by age group. We next describe how, based on the DIM data and COVID-19 case data, the assessment of vaccine effectiveness against different clinical endpoints (hospitalization, intensive care, death) is performed. While this method is used for a preliminary estimate of vaccine efficacy, population-based nonrandomized studies are able to provide more precise and detailed estimates under “real-world” conditions. In this context, we describe the hospital-based case-control study COViK, which is being conducted in collaboration with the Paul Ehrlich Institute (PEI). We discuss strengths and limitations of the abovementioned structures and tools. Finally, we provide an outlook on future challenges that may arise during the ongoing pandemic and during the transition phase into an endemic situation.

## Einleitung

Zu den Hauptaufgaben des Fachgebiets Impfprävention des Robert Koch-Instituts (RKI) gehören die Erhebung von Impfquoten und das Monitoring der Wirksamkeit von Impfungen. Mit der Einführung von Impfstoffen gegen COVID-19 im Dezember 2020 stand das Fachgebiet vor neuen Herausforderungen. Diese ergaben sich u. a. daraus, dass hierbei Impfstoffe zum Einsatz kamen und kommen, für die noch keine Erfahrungen in der breiten Anwendung vorlagen, die zunächst ausschließlich in Impfzentren bzw. durch mobile Impfteams verabreicht wurden und deren Langzeitwirksamkeit unbekannt war. Vor diesem Hintergrund war es notwendig, neue Strukturen und Instrumente zur Erhebung der Impfquoten und zur Ermittlung der Impfstoffwirksamkeit zu schaffen. Im Folgenden werden dazu exemplarisch das Digitale Impfquotenmonitoring (DIM), Auswertungen nach Impfstatus und die krankenhausbasierte Fall-Kontrollstudie zur Wirksamkeit der COVID-19-Impfung (COViK) beschrieben.

## Neue Erhebungssysteme für Impfungen in der Pandemie

### Grundlagen und Struktur des Digitalen Impfquotenmonitorings (DIM)

Mit dem in Deutschland etablierten Impfmonitoringsystem werden die empfohlenen Standardimpfungen regelmäßig hinsichtlich der Impfinanspruchnahme und zur Untersuchung von Impfeffekten in Auswertungsstichproben evaluiert. Kernstück des Routine-Impfmonitorings sind die quartalsweisen Abrechnungsdaten der Kassenärztlichen Vereinigungen (KVen), die dem RKI seit 2004 zunächst im Rahmen eines Projektes und seit März 2020 auf der Grundlage des Infektionsschutzgesetzes (IfSG) übermittelt werden. Diese Daten der Vertragsärzte liegen jedoch erst in großem zeitlichen Abstand zum jeweiligen Impfzeitpunkt vor und umfassen nicht die Gesamtbevölkerung. Um in einer Pandemie den erreichten Stand der Impfkampagne zu jedem Zeitpunkt einschätzen und die Wirksamkeit und Sicherheit von Pandemie-Impfstoffen im breiten Bevölkerungseinsatz fortlaufend bewerten zu können, werden aktuelle, impfstoffspezifische und vollständige Daten zur Impfinanspruchnahme benötigt. Die COVID-19-Impfkampagne begann in Deutschland außerhalb des Routine-Impfsystems in Impfzentren und mithilfe von mobilen Impfteams. Vor diesem Hintergrund reichte das bestehende System des Impfmonitorings für die besonderen Anforderungen der Pandemiesituation nicht aus und es wurden insgesamt 3 neue zusätzliche Meldesysteme etabliert (siehe nächster Abschnitt „Zusätzliche Meldeportale in der Pandemie“).

Das RKI hat beim pandemischen Impfmonitoring eine zentrale Funktion. Die tägliche Erfassung der Impfdaten in allen impfenden Stellen und die Übermittlung an das RKI haben ihre rechtliche Grundlage in der Coronavirus-Impfverordnung [5]. In dieser Verordnung sind u. a. alle Leistungserbringer von COVID-19-Impfungen festgelegt (siehe § 3) sowie die unterschiedlichen Wege der Datenübermittlung, die jeweils eigene Meldestrukturen erfordern, verbindlich vorgegeben (siehe § 4).

### Zusätzliche Meldeportale in der Pandemie

**Figure 1:**
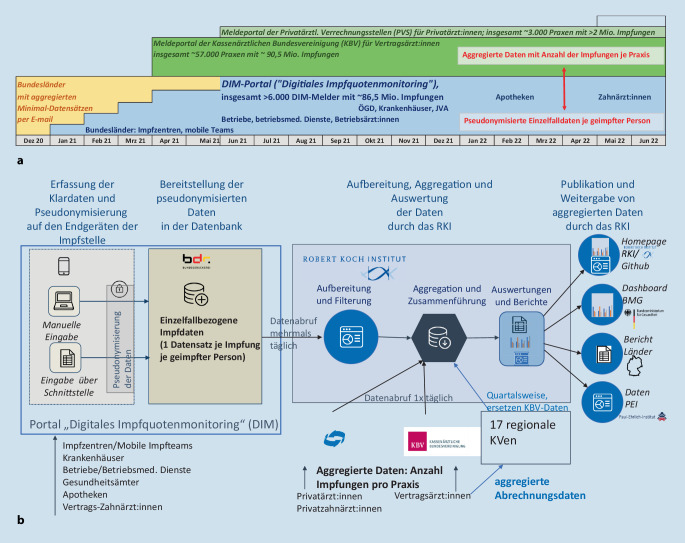

Das RKI hatte den Auftrag, für alle Impfstellen, die nur für die COVID-19-Impfkampagne eingerichtet wurden und nicht zum Routine-Impfsystem gehören, ein Erhebungssystem zum digitalen Impfquotenmonitoring (DIM-Portal) zu entwickeln. Dieses Meldeportal ist ein gemeinsames Projekt des RKI, der Bundesdruckerei Gruppe GmbH (Bdr) und der Accenture Dienstleistungen GmbH, finanziert durch das Bundesministerium für Gesundheit (BMG). Das RKI hat die Bdr mit der technischen Umsetzung und Accenture mit dem Projektmanagement beauftragt. Über dieses Portal können pseudonymisierte Einzelfalldaten übermittelt werden und es ist seit dem Start der Impfkampagne am 27.12.2020 einsatzbereit. Dazu gehören alle von den Bundesländern eingerichteten Impfzentren, die mobilen Impfteams sowie einige Krankenhäuser (seit 27.12.2020), Betriebe und Betriebsmedizin (seit Juni 2021), alle Krankenhäuser sowie Gesundheitsämter (seit Oktober 2021), Apotheken (seit Februar 2022) und die Zahnärzt:innen (seit Mai 2022). Diese Einrichtungen wurden nach und nach als Leistungserbringer in die Impfkampagne aufgenommen und sukzessive an das DIM-Portal angeschlossen (Abb. 1a).

Ein weiteres Meldeportal wurde notwendig, als im April 2021 der Start der COVID-19-Impfungen bei den Vertragsärzt:innen erfolgte. Für sie stellt die Kassenärztliche Bundesvereinigung (KBV) ein Portal für tägliche aggregierte Impfmeldungen zur Verfügung. Übermittelt wird hier lediglich die Anzahl der durchgeführten Impfungen nach Impfstoff und Impfserie sowie, getrennt davon, die Anzahl der Impfungen nach bis zu 4 Altersgruppen und Impfserie. In ähnlicher Weise, allerdings in einem Datenpaket mit Impfstoff, Altersgruppe und Impfserie, übermitteln die seit Juni 2021 beteiligten Privatärzt:innen ihre Daten über ein Portal der Privatärztlichen Abrechnungsstellen (PVS). Aus diesen beiden Portalen ruft das RKI täglich die Daten ab. Zusätzlich übermitteln die KVen quartalsweise höher aufgelöste aggregierte Impfabrechnungsdaten der Vertragsärzt:innen an das RKI, welche die KBV-Tagesdaten rückwirkend ersetzen. Erst wenn auch die pseudonymisierten einzelfallbezogenen Abrechnungsdaten aus allen KVen am RKI vorliegen und mit den über das DIM-Portal übermittelten Impfdaten zusammengeführt werden, sind vollständige individuelle Impfserien auswertbar und die routinemäßigen Auswertungen zu differenzierten Impfquoten nach Alter, Geschlecht, Risikogruppen und Region sowie zu Impfeffekten werden durchgeführt.

Das Gesamtsystem aus allen Meldewegen und die sich daraus speisenden Datenbestände bilden das Digitale Impfquotenmonitoring (DIM) für COVID-19-Impfungen am RKI. Eine Übersicht über die Datenflüsse gibt Abb. 1b.

### Verwendung der COVID-19-Impfdaten

Die Impfdaten erfahren ein hohes Maß an öffentlicher und politischer Aufmerksamkeit. Das RKI wertet die Daten täglich aus und publiziert sie auf unterschiedlichen Wegen: Auf der RKI-Homepage können die aktuellen Impfquoten je Bundesland in einer Karte abgelesen werden und es stehen kumulierte Zahlen der Impfinanspruchnahme und Impfquoten bundesweit und je Bundesland in einer Excel-Datei zur Verfügung [6]. Diese Daten werden auf dem Impfdashboard des BMG visualisiert (COVID-19-Impfdashboard [7]). Zusätzlich gibt es Datenübersichten zu jedem Impftag und aufgeschlüsselt nach Impfstoff, Impfserie, Altersgruppe sowie dem Ort (Land- bzw. Stadtkreis) der Impfstelle, die das RKI auf der Plattform des Internet-Hosting-Dienstes GitHub [8] bereitstellt. Auf dieser Plattform findet sich außerdem auch eine ausführliche Beschreibung der Datenquellen, der Aufbereitung der Daten, der Variablen sowie der Metadaten. Des Weiteren liefert das RKI mehrmals wöchentlich aggregierte Daten zum Impfgeschehen an jedes Bundesland zurück.

Die Auswertungen des RKI geben Trends der Impfquoten in der Gesamtbevölkerung, in den Altersgruppen, für die Daten verfügbar sind, sowie regional nach Bundesländern wieder. Es lassen sich daraus die Anzahl der Ungeimpften sowie der Impffortschritt bei Grundimmunisierung und Auffrischimpfung je Altersgruppe und Region ableiten und es können die Zahlen verimpfter und bereitstehender Impfstoffdosen je Impfstoff verglichen werden.

Die Impfquoten sind außerdem die Basis für die Bewertung der Wirksamkeit der Impfung: So werden z. B. die COVID-19-Inzidenzen in der geimpften und der ungeimpften Bevölkerung gegenübergestellt oder wird mit der sog. Screening-Methode der Anteil der Geimpften unter COVID-19-Fällen mit dem Anteil der Geimpften in der Gesamtbevölkerung verglichen. Weitere Auswertungen zur Impfstoffwirksamkeit mithilfe der Impfdaten differenzieren nach Alter, nach Impfstoff und nach Impfstoffdosis. Auch diese Analysen werden vom RKI regelmäßig zunächst in Wochenberichten und seit Juli 2022 in einem Monatsbericht publiziert und der Ständigen Impfkommission (STIKO) für die Entwicklung ihrer Empfehlungen zur Verfügung gestellt.

### Limitationen der Auswertung der COVID-19-Impfdaten

Die Aussagekraft der COVID-19-Impfdaten ist durch die geringe Granularität der Daten aus den Portalen der KBV und der PVS, die zusammen mehr als die Hälfte aller COVID-19-Impfungen umfassen, eingeschränkt. Übermittelt werden aus diesem Bereich nur Daten zum Alter in den Gruppen 5–11 Jahre, 12–17 Jahre, 18–59 Jahre und ab 60 Jahre, sodass keine Analysen nach feineren Altersgruppen oder nach Geschlecht vorgenommen werden können. So kann z. B. für Impfungen, die spezifischen Altersgruppen empfohlen sind – wie die zweiten Auffrischimpfungen für Personen ab 70 Jahren –, keine genaue Aussage zur Inanspruchnahme in dieser Gruppe getroffen werden. Eine Auswertung zum Geschlecht der Geimpften war erstmals mehrere Monate rückwirkend im Juli 2022 möglich, nachdem die über das KBV-Portal übermittelten Daten bis zum Impfdatum 31.12.2021 durch die KV-Abrechnungsdaten aus dem Jahr 2021 vollständig ersetzt werden konnten [1].

Des Weiteren fehlt die Zuordnung der Impfungen zum Wohnort der Geimpften (angegeben ist stattdessen der Ort der Impfung). Damit sind keine kleinräumigen aktuellen Analysen möglich und selbst die bundeslandbezogenen Auswertungen geben eher die Dichte der impfenden Stellen als die wirkliche Bundeslandimpfquote wieder. Da der Bezug der durchgeführten Impfungen in den Impfstellen eines Bundeslandes jedoch die Wohnbevölkerung ist, können rechnerisch auch Impfquoten > 100 % erreicht werden.

Eine weitere Limitation besteht in der fehlenden Zuordnung von Impfstoff und Altersgruppe in den KBV-Daten. Der Aufbau bzw. die Benennung von impfenden Stellen lag in der Verantwortung der Bundesländer ebenso wie die Anmeldung der einzelnen Impfstellen (einschl. Krankenhäuser) an das DIM-Portal. Da die Krankenhäuser keine spezifische Kennzeichnung im System bekamen, ist für das RKI nicht nachvollziehbar, welche und wie viele Krankenhäuser im Einzelnen angebunden wurden. Im DIM-Portal kann zwar jederzeit nachgemeldet werden, jedoch gibt es keine Nachmeldemöglichkeit über das KBV-Schnellmeldeportal. Diese Daten werden jedoch rückwirkend durch die KV-Abrechnungsdaten ersetzt.

### Schlussfolgerung zur Auswertung der COVID-19-Impfdaten

Die Impfquoten bilden eine wichtige Grundlage für die Evaluation der Umsetzung der Impfempfehlungen und für politische Entscheidungen zum Impfgeschehen in der Pandemie. Sie erlauben es, den aktuellen Stand und Fortgang der bundesweiten Impfkampagne einzuschätzen, und sind für die Bewertung der Impfstoffwirksamkeit von großem Nutzen. Analysen der Impfinanspruchnahme nach Zielgruppen und Region sind allerdings nur mit Einschränkungen möglich. Eher ungeeignet sind die bereitstehenden Daten wegen der fehlenden Granularität für die Bewertung der Impfstoffsicherheit durch das Paul-Ehrlich-Institut (PEI). Für weitergehende Analysen zu Impfeffekten, zur Dauer des Impfschutzes sowie für Subgruppenanalysen ist die Verbindung der DIM-Daten mit den KV-Daten unverzichtbar.

## Inzidenzen und Impfeffektivität: Auswertungen nach Impfstatus

### Überwachung der Impfeffektivität

Für die in Deutschland verwendeten COVID-19-Impfstoffe konnte in den Zulassungsstudien eine hohe Effektivität sowohl gegen eine Infektion mit SARS-CoV‑2 als auch gegen schwere Verläufe von COVID-19 gezeigt werden [2]. Die Zulassungsstudien boten jedoch keine belastbaren Erkenntnisse zur Frage, ob sich diese hohe Effektivität unter Realbedingungen in einer Bevölkerung bestätigt, die auch die in Zulassungsstudien z. T. ausgeschlossenen oder unterrepräsentierten Risikogruppen enthält, sowie zur Dauer des Impfschutzes oder zum Schutz der Impfung gegenüber Erkrankung durch die aufkommenden neuen Virusvarianten. Aus diesem Grund überwacht und bewertet das RKI als Teil der Evaluation der STIKO-Impfempfehlungen die Wirksamkeit der Impfung in der Bevölkerung Deutschlands seit Beginn der COVID-19-Impfkampagne. Dies erfolgt u. a. durch den Vergleich der COVID-19-Inzidenzen nach Impfstatus und die Abschätzung der Wirksamkeit mittels der sogenannten Screening-Methode nach Farrington [3].

Für die Berechnungen verwendet das RKI 2 Datenquellen. Zum einen sind dies die Daten aus dem DIM, welche eine Schätzung der Anzahl ungeimpfter, grundimmunisierter und geboosterter Personen in der Bevölkerung der Bundesrepublik in 4 Altersgruppen erlauben (s. oben). Zum anderen sind dies pseudonymisierte, fallbasierte Daten zu gemeldeten COVID-19-Fällen, die dem RKI aufgrund der Meldepflicht nach §§ 6 und 7 IfSG aus allen Bundesländern übermittelt werden: Als meldepflichtige Erkrankung muss jede laborbestätigte SARS-CoV-2-Infektion an die zuständigen Gesundheitsämter gemeldet werden, woraufhin die Gesundheitsämter weitere Angaben zur betroffenen Person, wie beispielsweise Alter, Schwere der Erkrankung (Symptome, Hospitalisierung, Betreuung auf einer Intensivstation, Tod) und Impfstatus, ermitteln. Die Fälle werden pseudonymisiert und mit den ergänzten Angaben an die zuständigen Landesstellen und von dort wiederum an das RKI übermittelt. Die Ergebnisse beider Auswertungen und deren Interpretation wurden zunächst wöchentlich, später monatlich in den Berichten des RKI publiziert und in Datentabellen zur Verfügung gestellt [9]. Diese informieren Öffentlichkeit, Presse und Politik.

### Inzidenzen nach Impfstatus

**Figure 2:**
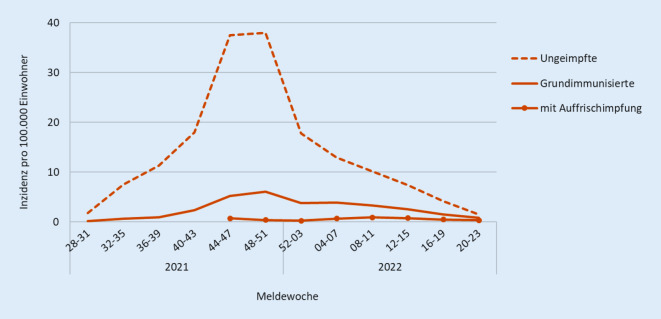

Um das Risiko für eine COVID-19-bedingte Hospitalisierung darzustellen und den Vergleich zwischen Ungeimpften und Geimpften zu ermöglichen, werden vierwöchentliche Inzidenzen mithilfe der beiden oben genannten Datenquellen berechnet. So wird beispielsweise für die Inzidenz unter den Ungeimpften ab 60 Jahren die Anzahl der nach IfSG übermittelten, aufgrund von COVID-19 hospitalisierten Fälle ermittelt, für die angegeben war, dass sie ungeimpft und mindestens 60 Jahre alt waren. Zu diesen Fällen wurde dann die Anzahl aller Personen in der Bevölkerung ins Verhältnis gesetzt, die zu diesem Zeitpunkt mindestens 60 Jahre alt waren und noch keine COVID-19-Impfung erhalten hatten. Entsprechend wird mit den grundimmunisierten und geboosterten COVID-19-Fällen bzw. der Bevölkerung verfahren (Abb. 2). Die Berechnungen werden für 3 Altersgruppen (12–17 Jahre, 18–59 Jahre, ≥ 60 Jahre) durchgeführt (nicht dargestellt).

Mit dieser Darstellung lassen sich Schlussfolgerungen zur Wirksamkeit der COVID-19-Impfung zum einen aus der Entwicklung der Inzidenz über die Zeit, zum anderen aus dem Vergleich der Inzidenz der Ungeimpften mit der Inzidenz der Grundimmunisierten bzw. Geboosterten ziehen. Der Anstieg der Hospitalisierungsinzidenzen sowohl unter Ungeimpften als auch Geimpften während der Dominanz der Delta-Variante spiegelte die erhöhte Schwere der Verläufe der Delta-Infektionen im Vergleich zur Alpha-Variante wider. Gleichzeitig wiesen die in diesem Zeitraum erhöhten Inzidenzen unter Grundimmunisierten auf eine leicht verminderte Wirksamkeit der Grundimmunisierung gegen Hospitalisierung hin. Die niedrigen Inzidenzen unter den Geboosterten zeigten wiederum, dass mit einer Boosterimpfung die vor der Delta-Dominanz beobachtete hohe Effektivität der Impfung gegen Hospitalisierung wiederhergestellt werden konnte. Sie zeigen aber auch, dass deren Wirksamkeit im Verlauf langsam sank.

### Schätzung der Impfeffektivität

**Figure 3:**
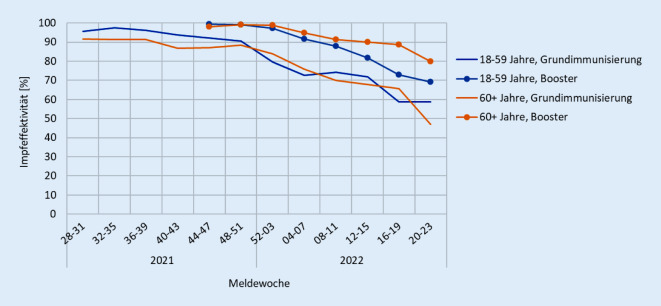

Die Screening-Methode nach Farrington erlaubt eine schnelle, mit geringem Aufwand verbundene Schätzung der Impfeffektivität, wenn der Impfstatus der aufgetretenen Fälle bekannt ist, für die Berechnung eines Odds Ratio in einem Fall-Kontroll-Ansatz jedoch Kontrollen fehlen [3]. Es wird der Anteil der Geimpften unter den Fällen zum Anteil der Geimpften in der Bevölkerung (d. h. der Impfquote) ins Verhältnis gesetzt und daraus die Impfeffektivität abgeleitet. Die Voraussetzung ist, dass Impfquoten zur Bevölkerung, der die Fälle entstammen, verfügbar sind. Damit lässt sich für die im DIM differenzierbaren Altersgruppen die Effektivität sowohl der Grundimmunisierung als auch der Auffrischimpfung für die in den Meldedaten nach IfSG enthaltenen klinischen Endpunkte (Hospitalisierung, intensivstationäre Betreuung, Tod) schätzen. Auch diese Analysen lassen bei Betrachtung der zeitlichen Verläufe sowie durch den Vergleich der Effektivität zwischen unterschiedlichen Altersgruppen oder zwischen Grundimmunisierung und Auffrischimpfung hilfreiche Interpretationen zu. So zeigte sich beispielsweise mit Auftreten der Omikron-Variante – den Ergebnissen internationaler Studien entsprechend [4] – eine sowohl nach Grundimmunisierung als auch nach Auffrischimpfung rasch abnehmende Effektivität gegenüber einer symptomatischen Infektion. Die hohe Effektivität der Grundimmunisierung gegenüber einer COVID-19-bedingten Hospitalisierung hielt während der Dominanz von Delta an und sank erst mit Auftreten von Omikron etwas ab (Abb. 3). Eine Auffrischimpfung, für die etwa ab Kalenderwoche 42/2021 relevante Bevölkerungsimpfquoten erreicht wurden, führte zu einem zusätzlichen Schutz, der sich auch mit der Dominanz von Omikron zunächst auf einem hohen Niveau hielt und langsam im zeitlichen Verlauf sank.

### Limitationen der Analysen zur Impfeffektivität

Die oben beschriebenen Analysen zur Überwachung der Impfwirksamkeit unterliegen zum einen den Limitationen, die aus den Einschränkungen der beiden verwendeten Datenquellen resultieren, zum anderen den Limitationen der Screening-Methode selbst. So sind mit den weit gefassten Altersgruppen aus dem DIM keine aussagekräftigen Auswertungen zu detaillierteren Altersgruppen möglich. Ebenso sind aufgrund der aggregierten Datenstruktur, aus der der zeitliche Abstand zur Impfung für Bevölkerungsgruppen nicht extrahiert werden kann, nur begrenzt Auswertungen zur Dauer des Impfschutzes möglich. Die insbesondere bei hohen täglichen COVID-19-Fallzahlen geringere Vollständigkeit bzw. Qualität der Meldedaten nach IfSG wiederum trägt neben einer generellen Untererfassung von meldepflichtigen Infektionen zur Unterschätzung der Inzidenzen in allen Impfstatusgruppen bei. Aus diesem Grund sollte bei dieser Auswertung der Vergleich der verschiedenen Impfstatusgruppen und nicht der absolute Wert der berechneten Inzidenz im Fokus stehen.

Darüber hinaus besteht die Möglichkeit, dass die Ergebnisse einer Verzerrung unterliegen, wenn die Datenvollständigkeit in den Impfstatusgruppen unterschiedlich ausgeprägt ist, wofür es jedoch keine Hinweise gibt. Geringe Fallzahlen beispielsweise für schwere Verläufe oder in den jüngeren Altersgruppen führen zu größeren wöchentlichen Schwankungen der Schätzwerte, sodass in diesen Fällen der Fokus immer auf den zeitlichen Verlauf der Werte gerichtet werden sollte.

Die Screening-Methode wiederum gelangt bei sehr hohen und sehr niedrigen Impfquoten an ihre Grenzen: Da sehr hohe Impfquoten bisher nicht erreicht sind, trägt dieser Umstand hauptsächlich dazu bei, dass es nach neuen Impfempfehlungen mehrere Wochen dauert, bis ausreichend hohe Impfquoten erreicht und damit belastbare Impfeffektivitätsschätzungen möglich sind. Schließlich muss beachtet werden, dass Impfquoten nicht verfügbar sind, wenn eine Impfempfehlung ausschließlich für Risikogruppen vorliegt. So wird beispielsweise die Impfeffektivität bei jüngeren Kindern unterschätzt, da den STIKO-Empfehlungen entsprechend überproportional viele Kinder mit erhöhtem Risiko für schwerere Verläufe geimpft sind, jedoch nur die Impfquoten für die Altersgruppe und nicht die entsprechende Risikogruppe verwendet werden können.

### Schlussfolgerung zur Analyse der Impfeffektivität

Unter Beachtung der genannten Limitationen bieten die Auswertungen zur Impfwirksamkeit eine zeitnahe und wenig aufwändige Möglichkeit, mit bereits verfügbaren Daten die Impfempfehlungen der STIKO zu evaluieren, die Öffentlichkeit, die Politik und auch die STIKO zu informieren und gegebenenfalls Empfehlungen abzuleiten. Die Ergebnisse zeigen auch bei unvorhergesehenen Änderungen der Lage (z. B. bei wechselnden Virusvarianten) mit nur geringer Verzögerung die Entwicklung der Impfeffektivität in der Bevölkerung. So konnte die abnehmende Effektivität der COVID-19-Impfung gegenüber einer symptomatischen Infektion ab dem Auftreten der Omikron-Variante beobachtet werden, jedoch bestätigten die Auswertungen gleichzeitig die hohe Impfeffektivität gegenüber einer Hospitalisierung oder noch schwereren Verläufen, welche bisher nur leicht absank.

## COViK: eine krankenhausbasierte Fall-Kontrollstudie zur Wirksamkeit der COVID-19-Impfung

Wie oben dargestellt, kann eine grobe Schätzung der Impfeffektivität unter Verwendung von Impfquoten und Falldaten nach Impfstatus durchgeführt werden. Um die Wirksamkeit von Impfstoffen jedoch im Detail zu analysieren, sind nichtrandomisierte Studien, wie z. B. Fall-Kontrollstudien, test-negative Studien (eine Variante des Fall-Kontroll-Designs, die ursprünglich zur Schätzung der Effektivität der Influenzaimpfung entwickelt wurde) oder Kohortenstudien, von großer Bedeutung. Im Frühjahr 2021 wurde am RKI in Zusammenarbeit mit dem PEI eine bundesweite, krankenhausbasierte Fall-Kontrollstudie zur Untersuchung der Wirksamkeit von COVID-19-Impfstoffen (COViK) etabliert, die durch das BMG gefördert wird.

**Figure 4:**
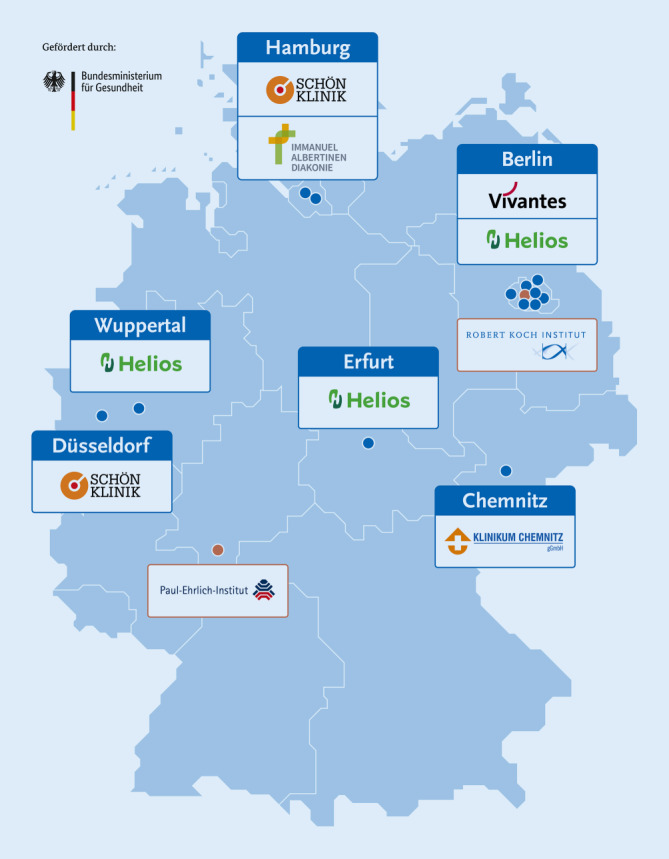

Deutschlandweit nehmen 13 Kliniken an der Studie teil. In Berlin besteht eine Kooperation mit dem Netzwerk der Vivantes Kliniken. Beteiligt sind die Kliniken Neukölln, Am Friedrichshain, Am Urban und Spandau sowie das Humboldt-Klinikum und das Auguste-Viktoria-Klinikum. Weitere Studienstandorte sind die Helios-Kliniken Berlin-Buch, Erfurt und Wuppertal sowie die Schön-Kliniken Hamburg-Eilbek und Düsseldorf, das Albertinen Krankenhaus in Hamburg und das Klinikum Chemnitz (Abb. 4 und Liste der Kooperationspartner in der Infobox 1).

Primäres Ziel ist die Ermittlung der Effektivität von COVID-19-Impfstoffen gegen Hospitalisierung infolge einer laborbestätigten SARS-CoV-2-Infektion bei Erwachsenen im Alter von 18 bis 90 Jahren. Weitere Ziele sind unter anderem die Bestimmung der Impfeffektivität bei Untergruppen von Personen, z. B. bei älteren oder vorerkrankten Personen, sowie die Bestimmung der Impfeffektivität gegen schwere Verlaufsformen (Behandlung auf der Intensivstation). Zudem sollen die Impfeffektivität von 2 oder mehr Impfstoffdosen und die Dauer des Impfschutzes bestimmt werden.

Im Verlauf der Pandemie traten bislang mehrere Virusvarianten (Alpha, Delta, Omikron) auf, die Ansteckungswellen verursachten. Die Varianten können unterschiedlich gut von den durch die Impfung generierten Immunzellen und Antikörpern erkannt werden, weshalb die Impfeffektivität für die einzelnen Wellen der Pandemie im Rahmen von COViK separat ausgewertet wird. Im Verlauf der Studie werden auch Langzeitverläufe (sog. Long COVID) bei geimpften und ungeimpften Personen charakterisiert. Hierfür werden in halbjährlichem Abstand telefonische Nachbefragungen der Teilnehmer:innen durchgeführt. Zusätzlich wird die Sicherheit der Impfstoffe in Hinblick auf potenzielle infektionsverstärkende Antikörper am PEI untersucht.

Die Patientenrekrutierung startete im Juni 2021 und erstreckt sich über einen Zeitraum von 2 Jahren. Es wird angestrebt, 1200 Fälle und 2400 Kontrollen in die Studie einzuschließen. Als Fälle kommen Patient:innen infrage, die wegen einer COVID-19-Erkrankung im Krankenhaus behandelt werden (Aufnahme‑/Einweisungs- oder Hauptdiagnose) und mittels PCR-Tests positiv auf SARS-CoV‑2 getestet wurden. Als Kontrollpatient:innen (sog. Kontrollen) werden in operativen Abteilungen pro Fall 2 Patient:innen rekrutiert, die hinsichtlich ihres Geschlechts, Alters, Zeitpunkts der Krankenhausaufnahme und Orts zum jeweiligen Fall passen. Zur Sicherung der Diagnose und Bestimmung der Virusvariante werden PCR-Tests und Sequenzierungen durchgeführt. Eine potenzielle SARS-CoV-2-Infektion bei den Kontrollen wird ebenfalls mittels PCR-Tests ausgeschlossen. Fall-Patient:innen wird bei Studieneinschluss eine Serumprobe zum Nachweis von Antikörpern abgenommen, gefolgt von einer weiteren Serumprobe nach 2–4 Wochen. Bei den Kontrollen dient die Testung auf Antikörper bei Studieneinschluss dem Nachweis von gegebenenfalls stattgehabten Infektionen, eine weitere Serumprobe ist nicht vorgesehen. Vorerkrankungen, Medikation, Krankheitsverlauf und Risikoverhalten werden detailliert erhoben. Somit hebt sich diese Studie von vielen anderen Impfstoffwirksamkeitsstudien dadurch ab, dass Informationen über die COVID-19-Erkrankung sowie mögliche Störgrößen (Confounder) sehr detailliert erhoben werden.

**Figure 5:**
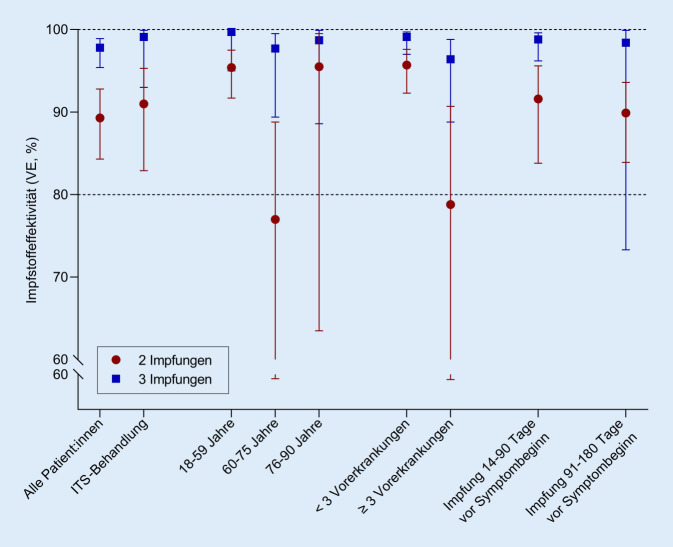

Im Frühjahr 2022 wurde eine im Protokoll geplante Zwischenanalyse zur Wirksamkeit während der Delta-Welle durchgeführt. Im Zeitraum vom 01.06.2021 bis zum 31.01.2022 wurden insgesamt 852 Teilnehmer:innen im Alter von 18 bis 90 Jahren in die Studie eingeschlossen, davon 244 COVID-19-Patient:innen mit schwerem Krankheitsverlauf und 608 Kontrollpatient:innen. 30 % der Fälle und 53 % der Kontrollen waren zweifach geimpft, 4 % der Fälle und 32 % der Kontrollen hatten eine dritte Impfung erhalten. 56 % der Studienteilnehmer:innen waren männlich, 44 % weiblich. Für die Grundimmunisierung wurden überwiegend mRNA-Impfstoffe verwendet (28 % der Fälle und 68 % der Kontrollen), Vektorimpfstoffe sowie Kombinationen der Impfstoffe kamen jeweils bei < 10 % der Studienteilnehmer:innen zum Einsatz. Die Boosterimpfungen erfolgten nahezu ausschließlich mit mRNA-Impfstoffen. Der überwiegende Teil der COVID-19-Patient:innen (79 %) war mit der Delta-Variante des Virus infiziert. Die Impfstoffeffektivität lag bei 89 % (95 % Konfidenzintervall (KI): 84,3–92,8 %) nach 2 Impfstoffdosen und bei 98 % (95 % KI: 95,4–98,9 %) nach einer dritten Dosis. Darüber hinaus wurden Subgruppenanalysen nach Altersgruppe, Schweregrad der Erkrankung (ITS) sowie Anzahl von Vorerkrankungen vorgenommen (Abb. 5). Die Impfung schützte vor schwerer Erkrankung mindestens 6 Monate lang (nach zweifacher Impfung: 90 % (95 % KI: 83,9–93,6 %), nach dreifacher Impfung: 98 % (95 % KI: 73,3–99,9 %)). Im weiteren Verlauf der Studie wird der Impfschutz über längere Zeiträume analysiert.

### Limitation der COViK-Studie

Da die einzelnen Pandemiewellen separat ausgewertet werden müssen, ist die Zahl der Studienteilnehmer:innen in einer Welle nicht immer ausreichend groß, um alle geplanten Subanalysen durchführen zu können. Durch die Rekrutierung von Krankenhauspatient:innen kann ein Selektionsbias entstehen. Zudem können nicht alle schwer kranken Patient:innen in die Studie eingeschlossen werden. Auch Patient:innen mit fulminantem Verlauf, die im häuslichen Umfeld versterben oder direkt auf die Intensivstation verlegt werden, werden in der Regel nicht erfasst, ebensowenig palliative und hochbetagte Patient:innen, die eine Krankenhauseinweisung ablehnen. Eine weitere Limitation betrifft die Teilnahmebereitschaft der Patient:innen. Insbesondere ungeimpfte Patient:innen, die auch andere präventive Maßnahmen ablehnen, nehmen seltener an der Studie teil als geimpfte Patient:innen. Da dies auf die Fälle als auch die Kontrollen zuzutreffen scheint, wird die Berechnung der Impfeffektivität hiervon jedoch nicht beeinträchtigt.

### Schlussfolgerung zur Zwischenanalyse der COViK-Studie

Die verwendeten Impfstoffe zeigten unter Realbedingungen eine hohe Wirksamkeit bezüglich der Verhinderung schwerer COVID-19-Erkrankungen unter der Delta-Variante. Ein guter Schutz vor schwerer, hospitalisierungspflichtiger Erkrankung wurde mit 2 Impfstoffdosen bereits erreicht, konnte aber durch eine Boosterimpfung nochmals deutlich gesteigert werden. Die Dauer des Impfschutzes sowie der Schutz vor neuen Virusvarianten und Folgeerkrankungen (z. B. Long COVID) sind Gegenstand der bis 2023 laufenden Studie.

## Ausblick

Zum Zeitpunkt der Erstellung dieses Artikels (Juli 2022) ist die epidemiologische Situation in Deutschland im Hinblick auf die Impfung gegen COVID-19 charakterisiert durch:

- eine deutlich sinkende Inzidenz,
- einen starken Rückgang der Anzahl der täglich verabreichten Impfdosen sowie
- eine durch die Omikron-Variante bedingte deutliche Reduktion der Impfwirksamkeit gegen leichte Erkrankungen, bei erhaltener Wirksamkeit gegen schwere Verlaufsformen.

Die Herausforderungen der kommenden Monate der Pandemie werden vor diesem Hintergrund u. a. dadurch bestimmt, dass einerseits eine Prognose mit Blick auf den Herbst nur sehr schwer möglich ist sowie andererseits mehrere Impfstoffhersteller die Markteinführung von variantenspezifischen Impfstoffen angekündigt haben, deren Wirksamkeit und damit Potenzial in der Pandemiebekämpfung wiederum vor dem Hintergrund der Unsicherheit der Prognose kaum abschätzbar sind. Dies alles zeigt, dass auf absehbare Zeit die in diesem Artikel vorgestellten Strukturen und Instrumente wichtig bleiben und erhalten werden müssen, um Impfquoten (ggf. auch mit variantenspezifischen Impfstoffen) erfassen und die Wirksamkeit unter sich möglicherweise verändernden Bedingungen (neue Varianten mit verminderter Wirksamkeit der Impfstoffe; nachlassende Schutzwirkung über die Zeit) zeitnah bestimmen und schließlich den Übergang in die endemische Phase überwachen und gestalten zu können.

### Infobox 1 Liste der Kooperationspartner der COViK-Studie

- *Robert Koch-Institut:* Anna Stoliaroff-Pépin, Caroline Peine, Tim Herath, Johannes Lachmann, Delphine Perriat, Achim Dörre, Andreas Nitsche, Janine Michel, Marica Grossegesse, Natalie Hofmann, Thomas Rinner, Tanja Meyer, Brigitte G. Dorner, Daniel Stern, Fridolin Treindl, Ole Wichmann, Thomas Harder
- *Paul-Ehrlich-Institut:* Sascha Hein, Laura Werel, Eberhard Hildt, Doris Oberle
- *Klinik für Innere Medizin – Pneumologie und Infektiologie, Vivantes Klinikum Neukölln und Spandau, Berlin:* Sven Gläser
- *Vivantes Netzwerk für Gesundheit GmbH, Direktorat Klinische Forschung & Akademische Lehre, Berlin:* Helmut Schühlen
- *Klinik für Innere Medizin – Infektiologie, Vivantes Auguste-Viktoria-Klinikum, Berlin:* Caroline Isner
- *Klinik für Pneumologie und Infektiologie, Vivantes Klinikum im Friedrichshain, Berlin:* Alexander Peric
- *Schön Klinik Düsseldorf, Interdisziplinäre Notaufnahme, Düsseldorf:* Ammar Ghouzi
- *Schön Klinik Hamburg, Zentrale Interdisziplinäre Notfallaufnahme und Clinical Decision Unit, Hamburg:* Manuel G. Burkert
- *Helios Klinikum Berlin-Buch:* Annette Reichardt
- *Albertinen Krankenhaus, Hamburg:* Matthias Janneck, Guntram Lock
- *Helios Klinikum Wuppertal: *Petra Thürmann
- *Helios Klinikum Erfurt: *Dominik Huster
- *Klinikum Chemnitz: *Thomas Grünewald
